# Rumen Fluid from Slaughtered Animals: A Standardized Procedure for Sampling, Storage and Use in Digestibility Trials

**DOI:** 10.3390/mps5040059

**Published:** 2022-07-13

**Authors:** Riccardo Fortina, Sara Glorio Patrucco, Salvatore Barbera, Sonia Tassone

**Affiliations:** Department of Agriculture, Forest and Food Sciences, University of Turin, 10095 Grugliasco, Italy; riccardo.fortina@unito.it (R.F.); salvatore.barbera@unito.it (S.B.); sonia.tassone@unito.it (S.T.)

**Keywords:** rumen fluid collection, slaughtered ruminants, rumen fluid storage, *in vitro* digestibility

## Abstract

Digestibility trials need a viable rumen fluid as *inoculum* to degrade feeds. The variability of rumen fluid depends on the animal’s diet, while its viability is greatly influenced by the sampling and handling procedures. In this article, we present a replicable protocol for sampling the rumen fluid from slaughtered animals for *in vitro* digestibility trials. A detailed list of the tools and a step-by-step standardized procedure for the collection, storage and the transportation of the rumen fluid from the slaughterhouse to the laboratory is presented. We also describe a digestibility trial for establishing the maximum storage time of rumen fluid from sampling to its use. The results show that the rumen fluid, collected and maintained according to the proposed protocol, can be stored and used from 30 to 300 min from sampling without significantly compromising the fermentative activity of the microbial population.

## 1. Introduction

The digestibility is an important parameter for the evaluation of the nutritive value of feeds. There are numerous *in vivo* and *in vitro* methods to measure the apparent or the true digestibility. The *in vitro* methods are cheaper and less time-consuming [1]; most of them utilize the rumen fluid (RF) as *inoculum*. Its microbial composition is largely depending on the diet fed to the animal, but the sampling and the storage procedure of the RF can influence the viability of rumen microorganisms and affects the repeatability of results [2,3].

Rumen cannulation is considered a reference method for collecting representative samples of RF [4,5]. However, the necessity of fistulated or cannulated animals to provide this *inoculum* raises several ethical and practical problems, e.g., the need for surgical facilities, constant care to avoid infections and the costs associated with the long-term maintenance of these animals. RF can also be obtained using an esophageal cannula, but this procedure causes considerable stress to the animal, and such samples are often contaminated with saliva [6].

A more ethically acceptable approach that reduces stress and alleviates the suffering of animals by avoiding an invasive procedure is the collection of RF at slaughtering. The RF collected at slaughter in controlled conditions has a limited difference from that sampled by other methods [7,8,9].

Many digestibility studies have used the RF collected at the slaughterhouse as *inoculum* [10,11,12,13], and a video of the sampling procedure is available online [14]. Additionally, the influence of storage time and temperature on the ability of rumen microorganisms to degrade NDF has been investigated by many authors [15]. However, in all of these studies [13,16,17], different procedures and storage times have been used. To date, a standardized sampling and storage procedure of RF from slaughtered animals has not been described.

The objective of this paper was to propose a standardized and replicable protocol for extracting sampling and handling the rumen content from the slaughterhouse to the laboratory. A digestibility trial was also performed to evaluate the viability of the RF after different storage times.

## 2. Experimental Design

Steps and materials of the standardized protocol to collect the rumen content from slaughtered animals and extract the RF are described in Section 4 (Procedure).

Since the distance between the slaughterhouse and the laboratory can be very variable, an experiment was conducted to verify if time of storage of RF from 30 to 300 min after the collection can affect the digestibility of feeds. The effectiveness of the RF has been tested by comparing the apparent, true and NDF digestibility.

## 3. Materials and Equipment

### 3.1. Collection of the Rumen Fluid at Slaughterhouse

The materials and equipment for collecting the RF at the slaughterhouse (see Section 4.1) are: (1) thermic bottles (as many as necessary; suggested volume: 500 mL) filled with hot water (40 °C), (2) thermal bag or portable cooler box, (3) thermometer, (4) plastic beaker, (5) colander, (6) knife and spatula, (7) gloves, (8) gown, (9) nylon socks, (10) helmet, (11) goggles, (12) facemask.

### 3.2. Use of the Rumen Fluid at the Laboratory

The materials and equipment for using the RF at the laboratory (see Section 4.2) are: (1) 500 mL graduated cylinder (2) plastic beaker filled with hot water (40 °C) (3) funnel (4) cheesecloth (5) CO_2_ gas bottles (6) pH meter (7) thermometer (8) gloves (9) lab coat.

### 3.3. Storage of the Rumen Fluid

RF can be stored into thermic bottles up to 300 min after collection without significant differences in average values of digestibility, as demonstrated in the experiment described in this paper (see Section 4.3). Normally, storage time depends on the distance between slaughterhouse and laboratory.

## 4. Procedure

### 4.1. Collection of Rumen Fluid at the Slaughterhouse

Before going to the slaughterhouse, fill thermic bottles with hot water (40 °C) and place them inside a thermic bag or portable cooler box; take all materials and tools described in Section 3.1, and leave to the slaughterhouse properly dressed.

**Figure 1:**
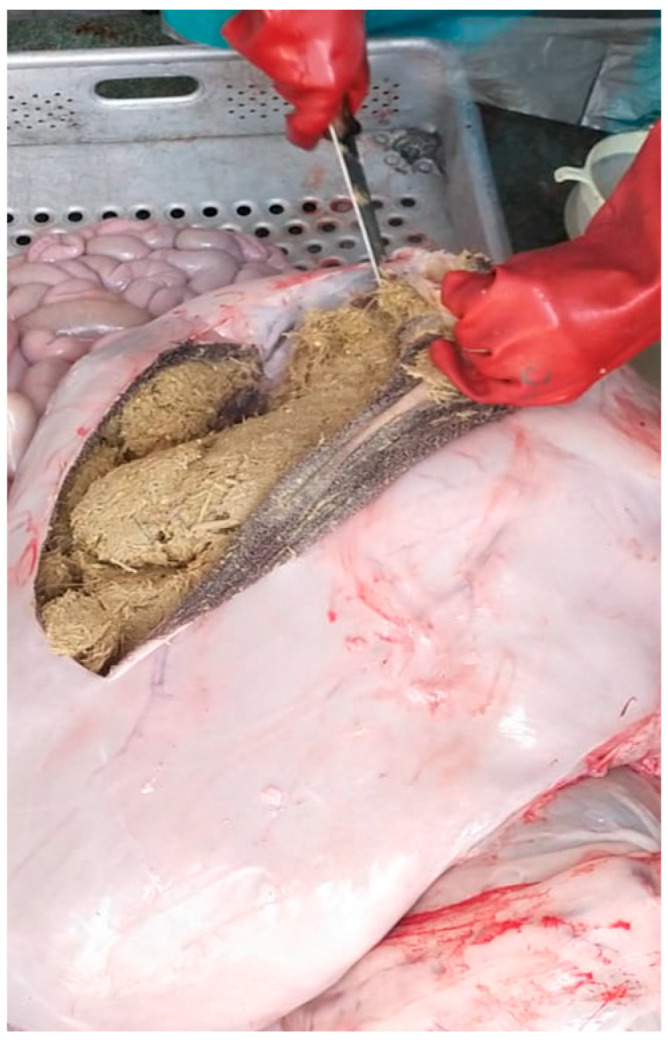
Sectioning of the rumen.

At the slaughterhouse, the interval between the animal death and the RF collection should not be longer than 15 min [13,17]. Place the rumen on a clean bench and section it lengthwise with a knife; carry out the next steps as quickly as possible and avoid an excessive contact of the rumen content with the air (Figure 1).

**Figure 2:**
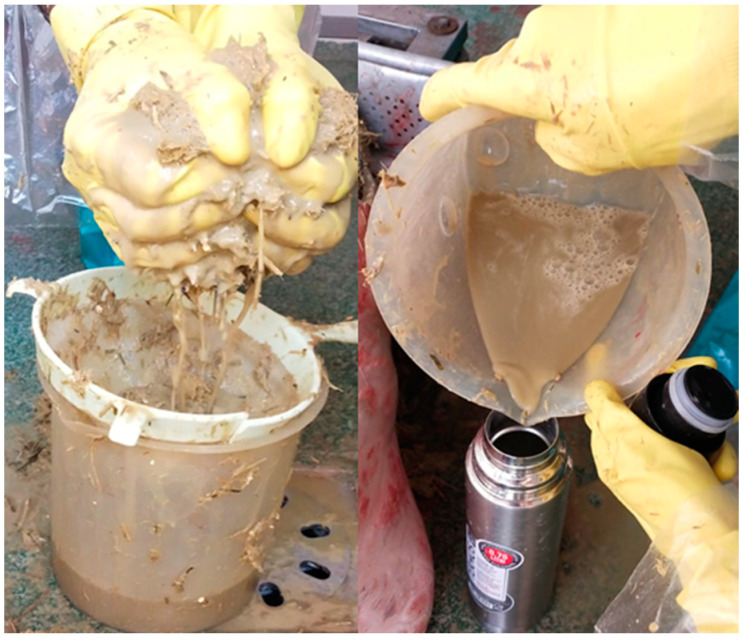
Squeezing and collection of RF at slaughterhouse.

**Figure 3:**
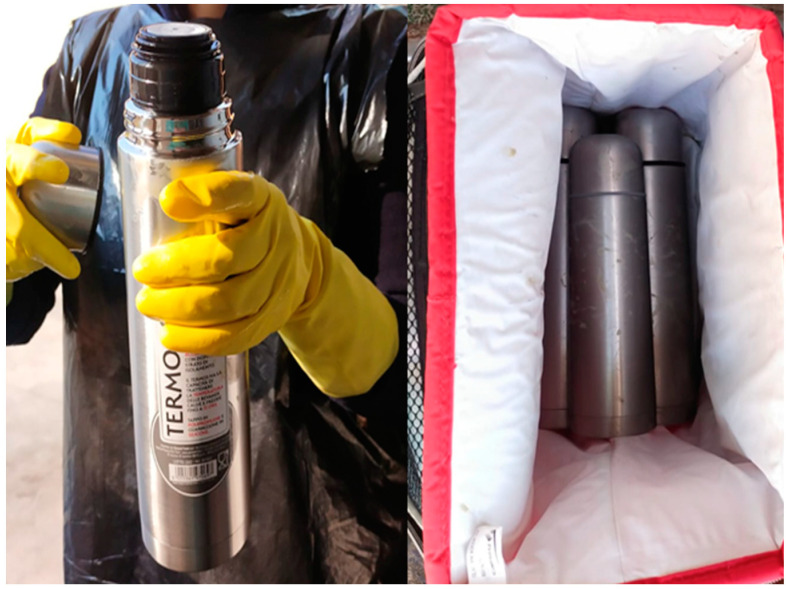
Thermic bottle with RF and its transport in thermic bag.

Sample 200–300 g of rumen content by hand and squeeze it into the plastic beaker, using the colander. Repeat this operation until approximately 500 mL of RF are collected; throw away the hot water from a thermic bottle and fill it up to the edge with the filtered RF; close immediately: no air should remain in the bottle (Figure 2). Proceed in the same way until all thermic bottles are full. Put them in the thermic bag and transport rapidly to the laboratory (Figure 3).

### 4.2. Use of the RF at the Laboratory

**Figure 4:**
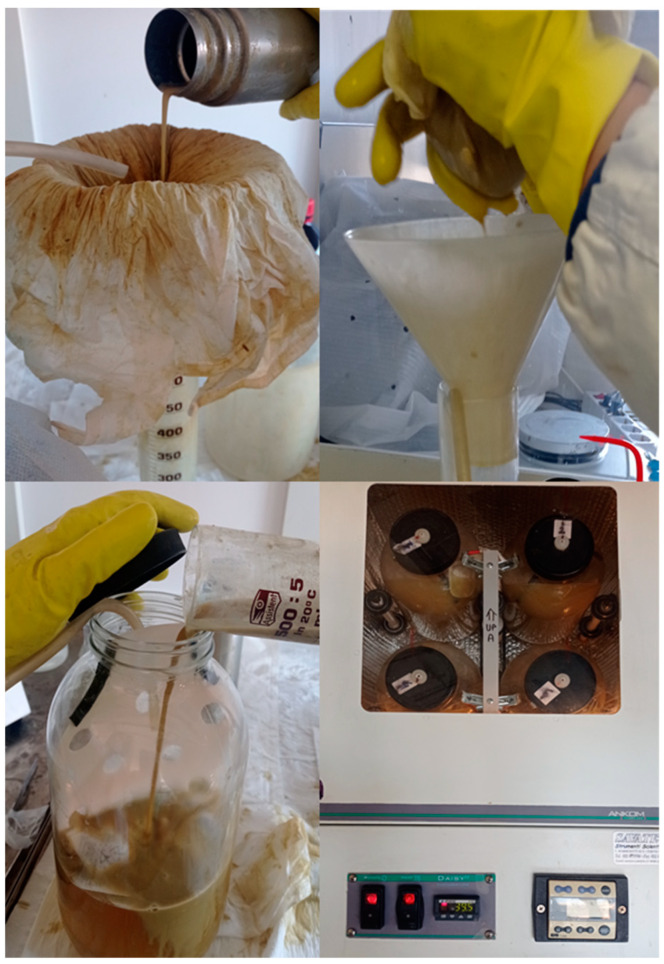
RF collection and incubation with the Ankom Daisy^II^.

On the arrival at the laboratory, place a 500 mL graduated cylinder into a plastic beaker filled with hot water (40 °C) under a hood. Put a funnel with the cheesecloth upon the cylinder; carefully open the thermic bottle inside a sink and pay attention to splashes: the pressure inside the bottles can cause violent release of RF. Pour the RF and squeeze it in the cylinder under CO_2_ flushing until the quantity for incubation is reached (with the Ankom Daisy^II^ Incubator, 400 mL/jar is needed); check pH and temperature of the fluid. Pour the filtered RF into a jar or a flask already containing the feed samples and the buffer solution; insufflate CO_2_ for 2 min. Close the jar or flask and start the digestion in the incubator at 39 °C (Figure 4).

### 4.3. Storage of Rumen Fluid and Digestibility Trial

The effectiveness of the RF after different storage times was determined using RF sampled at the slaughterhouse of the Department of Veterinary Medicine of the University of Turin (Italy) from 5 Limousine beef cattle (age: 14–16 months) during a 5-week period. All animals were fed with the same total mixed ration (TMR), chopped at 3 cm approximately, and made up of 7.5 kg concentrate (ground corn, beet pulp, soybean meal, bran, minerals and vitamins), mixed hay and straw; estimated DMI was 11 kg/day. A total of 5 RF were collected (one each week), following a standardized procedure (see Section 4.1). The RF of each animal was treated in the same way. Each RF sample was stored in 4 thermic bottles and used as *inoculum* for digestibility trial after 30 (T30), 90 (T90), 180 (T180) and 300 (T300) min from sampling, following a standardized procedure (see Section 4.2). The influence of RF storage time was tested by measuring the apparent, true dry matter and NDF digestibility (ADMD_AD_^II^; TDMD_AD_^II^; NDFD_AD_^II^) of 6 feeds (corn meal, soybean meal, wheat bran, beet pulp, mixed hay, wheat straw) and a total mixed ration (TMR). Their chemical composition is reported in Table 1.

Digestibility was performed with the Ankom Daisy^II^ Incubator (AD^II^, Ankom Technology Corporation Fairport, Macedon, NY, USA), a thermostatically controlled chamber (at 39 °C) containing 4 rotating digestion jars that allows to test different storage times of the same RF; the incubation time was 48 h.

ADMD_AD_^II^, TDMD_AD_^II^ and NDFD_AD_^II^ were calculated following the procedure described by Tassone et al. [11]. Briefly: to each of the 4 jars was assigned the same *inoculum* (run) after different waiting times from sampling (30, 90, 180, 300 min). Before the beginning of the incubation (30 min), feeds and TMR samples (0.5 g ± 0.05) weighted in triplicate into F57 bags (25-micron pore size; Ankom Technology Corporation Fairport, Macedon, NY, USA) were inserted into the jar filled with 1600 mL of buffer solution heated to 39 °C. In the meantime, the RF was filtered as described in Section 4.2, and 400 mL were added to the jar. The same procedure was repeated for each jar at different times from sampling.

At the end of the incubation (48 h) the bags were removed from each jar, rinsed thoroughly with cold tap water, and placed in a 50 °C forced-air oven to dry for 24 h. The bags were weighed and then analyzed for NDF with the Ankom200 Fiber Analyzer (Ankom Technology Corporation, Fairport, USA).

*In vitro* digestibility was calculated as follows:

ADMD_AD_^II^ (% DM) = 100 × (DM_0h_ − DM_residue_)/DM_0h_

TDMD_AD_^II^ (% DM) = 100 × (DM_0h_ − NDF_residue_)/DM_0h_

NDFD_AD_^II^ (% NDF) = 100 × (NDF_0h_ − NDF_residue_)/NDF_0h_

where:

DM_0h_ (%) = dry matter *ante* incubation
DM_residue_ (% DM) = dry matter *post* incubation
NDF (% DM) = neutral detergent fiber *ante* incubation
NDF_residue_ (% NDF) = neutral detergent fiber *post* incubation.

The temperature and pH of RF after different storage times (30, 90, 180, 300 min) before incubation and the temperature and pH of the buffered RF at the end of the incubation were measured.

The results were analyzed with SAS 9.4 software [18]. It used a covariate model fitting a linear regression to relate storage time as a continuous variable (30 to 300 min) to digestibility results. The 7-level feed classification variable was used in the covariate model to identify group membership to isolate the effect of the continuous variable. In our case, the maximum efficiency of the storage method would occur with a zero correlation between storage time and digestibility, indicating a constant efficiency of the RF.

The variability of digestibility was analyzed in order to verify the reliability of the rumen fluid storage method and the incidence of different feeds. The Standard Deviation (SD) calculated for each “feed × run × storage time” combination was used as dependent variable. A GLM with a bifactor model with interaction (feed and storage time) was used to compare each feed the variability at different storage times. Comparisons were made using the Tukey’s test adjusted for multiple comparisons.

## 5. Results and Discussion

Table 2 shows average values of *in vitro* apparent (ADMD_AD_^II^), true (TDTD_AD_^II^) and NDFD (NDFD_AD_^II^) digestibility of samples.

The results show that all feeds were digested in a similar way despite the different storage times. The results demonstrate that the rumen content, collected and maintained at the conditions previously described, can be used from 30 to 300 min from sampling without significantly compromising the digestibility of the feeds used in the trial.

The influence of storage time and temperature on the ability of rumen microorganisms to degrade NDF has been examined by other authors [15]. They reported that, within-day delays up to 6.5 h between the time of collection of the rumen *inoculum* and the beginning of the incubation, no effects were observed on the *in vitro* digestion of NDF when RF was maintained under anaerobic conditions at 39 °C. Some authors [7,15,19,20] have demonstrated that RF, or mixed ruminal microorganisms, can also be stored at low temperature without negative effects on gas production and some fermentation parameters.

The analysis of covariance, considering the feed as a fixed parameter and the storage time as a continuous parameter, clearly showed the different digestibility of the parameters between the feeds but, more interestingly for the purposes of the proposed method, a non-significant variation in the measured parameters over time.

**Figure 5:**
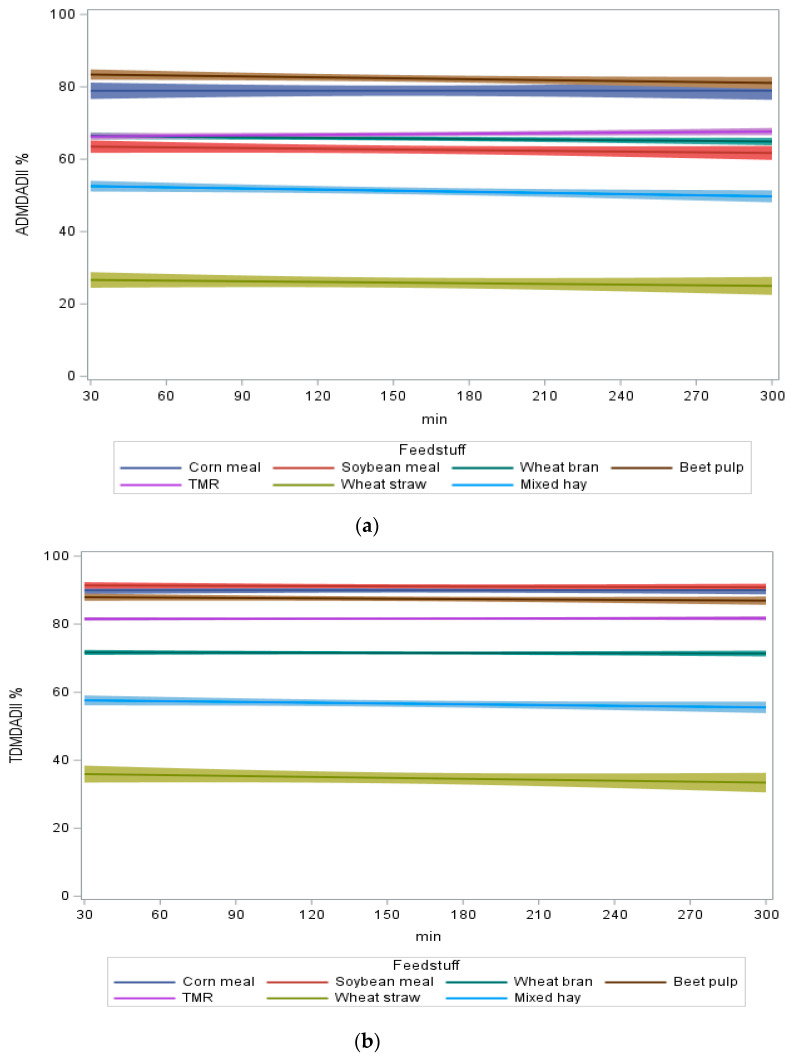

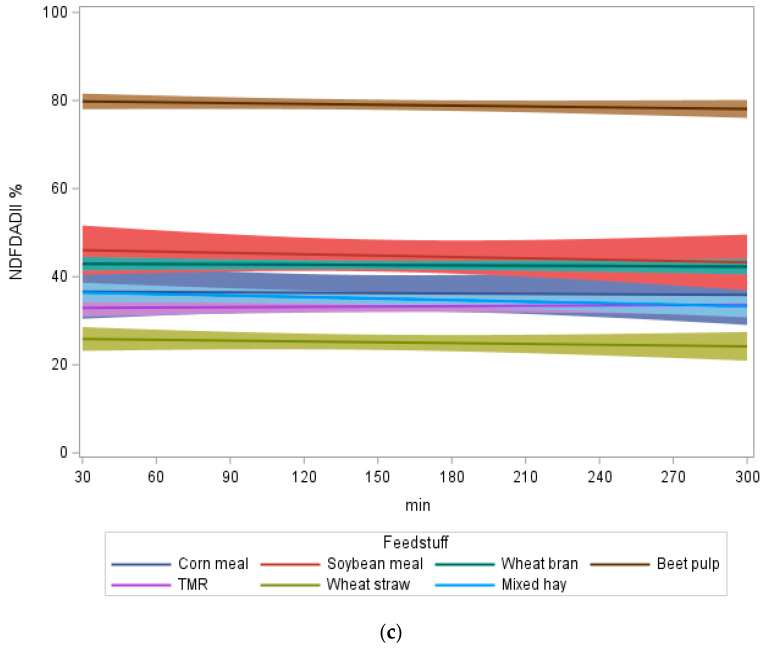
(**a**) Apparent dry matter digestibility (ADMD_AD_^II^) of different feeds and TMR incubated with RF after different storage times with the mean value confidence limits. (**b**) True dry matter digestibility (ADMD_AD_^II^) of different feeds and TMR incubated with RF after different storage times with the mean value confidence limits. (**c**) NDF digestibility (NDFD_AD_^II^) of different feeds and TMR incubated with RF after different storage times with the mean value confidence limits.

Figure 5a–c clearly shows how the storage time of the RF does not affect the effectiveness of the analysis with the Ankom Daisy^II^.

Thus, the proposed method allows the RF to be used between 30 and 300 min, when properly stored in a thermic bottle, without any change in the digestibility of the measured parameters.

The variability (SD calculated for each “feed x run x storage time” combination) for ADMD_AD_^II^ and TDMD_AD_^II^, among the combination of feed and storage time, was not significantly different. The ADMD_AD_^II^ varied between 0.67% (wheat bran at 30 min) and 3.59% (corn meal at 90 min). The TDMD_AD_^II^ varied between 0.49% (wheat bran at 30 min) and 2.74% (wheat straw at 300 min).

NDFD_AD_^II^ showed a greater variability (Table 3). In agreement with other authors [21], the two least fibrous feeds (corn and soybean meal) showed greater and more significant variability than the other feeds and the TMR; however, within each feed, the variability at different storage times was never significantly different.

The pH of RF decreases during the storage from 5.90 (30 min) to 5.40 (300 min) in agreement with other authors [22]. After 48 h of incubation, the pH of the buffered RF ranged from 6.27 to 6.19. The temperature of RF was 33.7 °C after 30 min of storage and increased to 35.5 °C after 300 min. These small variations in pH and temperature do not seem to have affected the effectiveness of the ruminal fluid in digestibility at different storage times. During incubation, the temperature reached an average value of 38.5 °C.

## 6. Conclusions

The reduction in the RF variability is a fundamental step in digestibility trials; a standardized sampling and storage procedure from the slaughterhouse to the laboratory can reduce its variability and assure the repeatability and the comparison between trials.

When properly collected and stored, the RF maintains its fermentative activity for a long period (up to 300 min) without significantly compromising the feed digestibility.

**Figure 6:**
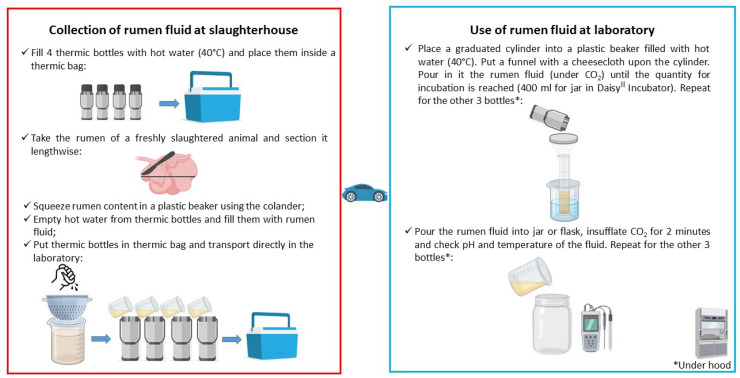
RF collection and storage procedure for *in vitro* digestibility analyses.

Figure 6 and Table 4 summarize the RF collection and storage procedures from the slaughterhouse to the laboratory for *in vitro* digestibility analyses, which can be used by researchers as protocol when they collect RF from slaughtered cattle. The experiment on storage times demonstrates that it is possible to collect and use the RF also when the slaughterhouse is far from the laboratory without compromising the results of the trials.

## Figures and Tables

**Table 1:** Composition of TMR and feeds (%DM).

	DM	Ash	CP	EE	NDF	ADF	ADL	NFC
Corn meal	87.0	1.4	9.3	4.7	12.3	4.5	1.0	72.3
Soybean meal	90.9	7.0	51.5	1.5	11.0	7.3	1.0	29.0
Wheat Bran	87.0	8.8	17.0	4.6	43.2	13.4	3.6	28.4
Beet pulp	90.1	5.3	9.1	0.7	53.9	28.8	2.6	31.0
Mixed hay	90.7	6.6	8.3	2.2	60.5	37.9	4.3	22.4
Wheat Straw	87.8	7.1	4.9	1.8	72.5	40.1	11.2	13.7
TMR	86.5	7.5	14.8	5.7	23.8	11.0	1.2	48.1

DM: dry matter; CP: crude protein; EE: ether extract; NDF: neutral detergent fiber; ADF: acid detergent fiber; ADL: acid detergent lignin; NFC: non-fiber carbohydrates. NFC = 100 − (Ash + CP + EE + NDF).

**Table 2:** *In vitro* digestibility of feeds used to assess the effect of RF storage time (N = 72).

	ADMD_AD_^II^	TDMD_AD_^II^	NDFD_AD_^II^
	Mean ± SD	Mean ± SD	Mean ± SD
Corn meal	78.9 ± 6.01	89.9 ± 2.71	36.3 ± 13.54
Soybean meal	62.7 ± 4.63	91.1 ± 2.32	44.8 ± 13.47
Wheat bran	65.7 ± 2.58	71.5 ± 1.93	42.7 ± 3.90
Beet pulp	82.4 ± 4.01	87.5 ± 2.68	79.0 ± 4.47
TMR	66.9 ± 2.45	81.6 ± 1.35	33.2 ± 4.91
Wheat straw	25.9 ± 5.95	34.8 ± 6.29	25.1 ± 5.51
Mixed hay	51.3 ± 4.09	56.7 ± 3.73	35.0 ± 5.60

ADMD_AD_^II^: apparent dry matter digestibility (%DM); TDMD_AD_^II^: true dry matter digestibility (%DM); NDFD_AD_^II^: NDF digestibility (%NDF); TMR: total mixed ration.

**Table 3:** Average standard deviation of NDFD_AD_^II^ of different feeds at different storage time (MSE = 7.98, N = 130).

Feeds	Storage Times			
	30′	90′	180′	300′
Corn meal	13.6	7.3	7.5	7.6
Soybean meal	3.9	3.7	7.4	5.4
Wheat bran	1.0	3.8	2.2	1.2
Beet pulp	2.6	3.3	4.5	3.5
TMR	3.1	2.9	2.4	3.4
Wheat straw	1.2	2.5	2.0	5.2
Mixed hay	3.3	1.7	2.9	2.0

No significant differences were found among storage time intra-feed.

**Table 4:** Rumen fluid collection and use procedure for *in vitro* digestibility.

**1**	**Materials and equipment preparation**
1a	For extraction of RF at slaughterhouse: (1) thermic bottles filled with hot water (40 °C), (2) thermal bag or portable cooler box, (3) thermometer, (4) plastic beaker, (5) colander, (6) knife and spatula, (7) gloves, (8) gown, (9) nylon socks, (10) helmet, (11) goggles, (12) facemask. For collection of RF at laboratory: (1) 500 mL graduated cylinder (2) plastic beaker with hot water (40 °C) (3) funnel (4) cheesecloth (5) CO_2_ gas bottles (6) pH meter (7) thermometer (8) gloves (9) lab coat (10) nylon socks.
1b	Fill the thermic bottles with hot water (40 °C) and place them inside a thermic bag or portable cooler box and take all material prepared at point 1.
**2**	**Collection of rumen fluid at slaughterhouse**
2a	Take the rumen of the slaughtered animal (15 min from death) and place it on a clean bench. Record animal code.
2b	Section the rumen (ventral sac) lengthwise with a knife. Record time and report it to the lab operator.
2c	Squeeze rumen content in plastic beaker using the colander.
2d	Empty hot water from thermic bottle and fill it with homogenized RF; avoid the presence of air and close it immediately. Proceed in the same way with the other bottles; perform as quickly as possible.
2e	Put thermic bottles in the thermic bag and transport directly to the laboratory as quickly as possible.
**3**	**Use of rumen fluid at laboratory**
3a	Place a 500 mL graduated cylinder into a plastic beaker filled with hot water (40 °C) under a hood.
3b	Put a funnel with a cheesecloth upon the empty cylinder.
3c	Carefully open the thermic bottle, avoid RF splashes; pour it in the cheesecloth upon the cylinder under CO_2_ and squeeze until the quantity for incubation is reached (400 mL for jar in Daisy^II^ Incubator). Perform as quickly as possible.
3d	Pour RF into jar or flask within 300 min from the collection, check pH and temperature of the fluid insufflating CO_2_ for 2 min. Perform as quickly as possible.

## Data Availability

Data are available in a publicly accessible repository.
